# Supplementary material for: Estimating the timing of multiple admixture events using 3-locus linkage disequilibrium
Source: PLoS Genet. 2022 Jul 15;18(7):e1010281. doi: 10.1371/journal.pgen.1010281 (PMC9342778; doi:10.1371/journal.pgen.1010281)
Supplement: Attachment — Submitted filename: Response to Reviewers.docx [file pgen.1010281.s001.docx]

**We thank both reviewers for their careful consideration of our work. Below we reproduce the editor’s and reviewers’ comments in full and we intersperse in bold our responses.**

Editor comments

(1) It would be helpful to make clear in the Introduction that this new technique is designed to detect multiple admixture pulses involving two sources, rather than (e.g.) involving three distinct sources.

**We modified the text according to the suggestion:**

**“We derive an equation that describes the decay of three-locus LD under an admixture history with multiple waves of migration from two source populations.”**

(2) Following from this, the 1000 Genomes applications were a bit unclear, given you analyse populations that are admixed from three sources. For example, what do you mean by "with Yoruba as reference"? I think you are assuming two pulses of admixture from a non-African source into an African one for these analyses? If so, it is not clear why this makes more sense than considering two pulses of African admixture. Also, what is the "non-Yoruba" source here -- is it an admixed Native/European source? It would be helpful to explicitly spell out what you used as surrogates for the admixing sources 1 and 2 here.

**We realize that these analyses were not explained in sufficient detail in the original version of the manuscript. We now write: “*We fitted a two-pulse model to the Mexican weighted LD surface (Fig \ref{ASH_MXL}) with Yoruba as the first source population and the other population being modeled as a missing source population, as previously described. The missing source population (non-Yoruban) represents an unknown admixed European and Native American population. This model was chosen to mimic the previous analysis of reference (30) which used a similar model to approximate continued gene-flow from Europeans and Native Americans*”.**

(3) Your paper figures are not likely to be very intuitive for most readers, including us. What are the lines in Figure 6 and 7, for example? Are these representing the best-fitting model? Most readers are likely to be more interested in plots like Figure 5, where you show estimation accuracy. But why is this only shown for T1 here, and not for T2? Readers might also be interested in what happens when the admixture proportions fixed in your model are inaccurate. While this is mentioned as a potential issue, at what point does it break down?

**Figures 6, 7 (now numbers 7, 8): yes, indeed it is a best-fitting model. We added the corresponding comment in the legend.**

**Figure 5: we extended the figure with a second panel showing the accuracy of T2 estimates, and a third panel showing Alder estimates of admixture time.**

**We also added a new figure (Figure 6) in order to show the effect of admixture proportion misspecification.**

(4) Detailed theory and applications involving multiple pulses of admixture, or specifically admixture between two sources followed by additional admixture from another source, have been described previously (Hellenthal et al 2014, Science 343:747; Ni et al 2018, Heredity 121:52), yet you do not reference these here. I appreciate your approach is different and has novelty, but it might be worth citing these in the Introduction.

**We are thankful for pointing these papers to us. We included the relevant references in the introduction.**

First reviewer

1) It is not clear whether the model choice (one/two/continuous waves of admixture) is a decision to be made a-priori by the operator or whether that appears from the results (eg. in Figure 6, should one look at the green portion of the plot to find out which are the best parameters along the Y and X axes?)

**The theory developed in the manuscript can be applied to any migration scenario, and theoretical LD surfaces can be calculated with our method. However, parameter estimation is possible only for two-pulse migration scenarios with our current software implementation.**

**We also improved and extended legends and captions of the figures to further help the reader in this regard. Figure 6 (now number 7) shows the 3-LD surface estimated from real data, and the level lines correspond to the best-fitting model.**

2) Although the ALDER output is somewhat used (Fig 4) as a reference for their novel results, it would be desirable to have a more clear benchmarking to assess whether A) the novel method is capable of discriminating between one, two or continuous admixture scenarios and B) by adding the ALDER (or MALDER) performance to figure 5, to what extent is the new approach better/comparable in retrieving admixture dates from the simulated data?

A) **The current implementation of our method does not apply for the inference of continuous migration. One pulse migration can be discriminated from a two pulse scenario if the estimated value of T_1 between the admixture events is small. We hope that the new extended Figure 5 helps to illustrate this.**
B) **We add figure 5C in order to show ALDER estimates of admixture time for different scenarios, and add the corresponding discussion in the text.**

Second reviewer

1.) Reference to equations in text:

I noticed two instances where the equation referred to in the text does not seem to match the equation being used in the analysis. I could be entirely mistaken here, but I thought I’d point them out just to be safe.

1.1) References to equation 8: Equation 8 is mentioned twice in the paper as the model used for two-pulse admixture (Line 212; Lines 225-227). However when inspecting this equation in the methods it appears to be a two-locus model rather than a three-locus model (only has a single genetic distance parameter d and no second d’ parameter). Further supporting this interpretation, the line preceding equation 8 (line 134) explicitly states that it is an “expression for the two-point covariance function”.

My question is did you mean to use the two-locus model here (eq 8) or is this supposed to be equation 7? (Eq 7 appears to be the 3-locus form)

**Thank you for noticing this. We fixed the equation numbers throughout the text.**

1.2.) References to equation 2: From the results section (lines 237-239 page 14) it seems like the model being tested in figures 1 and 2 is a two-pulse admixture model (Eq 7 or eq 8 depending on the answer to the above point). However in the figure legends of figures 1 and 2 (and on line 241) it is stated that the contours in these figures come from equation 2. Is this correct? I don’t see any parameters for admixture timings, or proportions (the variables being explored in the plots) in equation 2 so I am unclear how this would work.

**Thank you for noticing this. We fixed the equation numbers throughout the text.**

2.) Issues with figures:

2.1.) Figures 1-3:

While the message of these plots regarding how well theory and simulations line up under different admixture scenarios is nicely illustrated, I initially found it difficult to determine what exactly is being plotted in these figures for the following reasons:

i.) No axis labels: The heatmaps lack any x and y axis labels making it unclear at first glance what is being plotted. Please provide axis labels.

**We added axis labels.**

ii.) Parameter labels where axis labels expected: The grid labels describing the parameters for a given simulation (e.g. T1=0, T2=10 for figure 1) fall where I would expect the missing x and y axis labels to be (bottom and left sides of a plot) leading to further confusion. Maybe separating these grid labels from the plots using a dividing line, or placing them to the right and top of the plot would help to avoid confusion.

**We placed grid labels to the right and top of the plot.**

iii.) No scale of heatmap: There is no scale provided for the heatmaps meaning the colours are hard to interpret. Please provide a scale bar.

**We added scale bars to each figure. We hope it will help with the readability of the figures.**

2.2) Figure 4:

This figure needs a few adjustments to improve legibility I recommend the following tweaks:

i.) Axis labels: Please label the x and y axes in the plot for clarity. The axes are currently described in the figure legend but readability would be increased by labelling the axes directly.

ii.) Plot key: The key in the figure could be cleaned up by reordering it into three columns grouped by T2 values (i.e. First column is all T2=2, second is all T2=5 and third is all T2=10) to improve figure legibility. This way all reds, all greens and all blues are together and the reader will understand that the colour scheme refers to the T2 values intuitively. This will also make the message of the plot clearer.

iii.) T1 values ambiguous: Currently there is no way to distinguish which lines belong to which value of T1 as the colours of each line within a T2 value group are identical. This could easily be solved by using different line types for each value of T1 (i.e. solid lines for T1=10, dashed lines for T1=5 and dotted lines for T1=10) or different shades of the grouping colour (i.e. T1=0 is light, T1=5 is medium, T1=10 is dark).

**We redesigned the figure and its legend according to all the suggestions.**

2.3.) Figures 6-7: Labelling the scale bar here may be helpful to readers.

**We added the scale bar labeling, and extended the captions to help easier understand these figures (now numbers 7-8).**
